# Supplementary material for: Chromothripsis during telomere crisis is independent of NHEJ, and consistent with a replicative origin
Source: Genome Res. 2019 May;29(5):737–49. doi: 10.1101/gr.240705.118 (PMC6499312; doi:10.1101/gr.240705.118)
Supplement: Supplemental Material [file supp_gr.240705.118_Supplemental_file_1.zip › contigs/annotated_contigs/DB113/contig.3.DB113_length_996_mean_cov_17.6275100402.docx]

**DB113_length_996_mean_cov_17.6275100402**

```
CTGAGAATGACCCCACTGTGGAGGTGTGTCATCTTGGGGTGGCACCTTTACTCTGGGCATACTGCTGGGCAGGATGGTGCTGGGGCTGT
 >chr16:67922154-67922585 + E=3e-244
ATACCCCAGAAGTCTTCCAGAGCTGGTCTTGGATGGCTAGGAGCAGGGGCCTGGCTGTTCTGGCTGGCCGTCAGGGAGATTACCATCCT

CCTGCTGTGGCCTACTTTTCCCCTGGTTTTTGCTCTCTAAGTCCCTACTTTTCCATGGAATAGAGCGGGGCTTGGGGAGGGGGGAAAGA

ATCTGCTCTCTGGCTAGGGGCTTAAGGGCTTTGTGTGTTTTATCCTAGAGCTGGAGGAGAGGGGTGGGCTGATAGCCAAGCTGGCTGGG

CCCTGGGCACAGCTGCCCAGGCCAGAGCATCATGGTGATGGTGTTTCTCACATTTGCAGCACCGTGGACAAG|GAG|CCCAGACCAGCA
                                                                         >chr16:6792268
CATAGTAAGGGTTCAATAAATATTTGTTAATTAATGCGCTGAATGTCTGTAAAACAGGAGTGTTGTGTAGATTTCTGAGTGCCATCAAC
9-67922799 + E=3e-54
CCTGGA|GCCTGGAGCATGACCCTGAGATCGTGACACCACTTGAGTGGAATTTTCCATGTTCCTTTTTACCTCTAATTTGGATCTTTTT
        >chr16:67918272-67918675 + E=2e-230
GTTTTTGAAAAACATTGAGAAATTCAATTAAATGCTTTTGGAATAAAATGGAGTATGTGTGTGCTTTTGGTTTGTCTTTGGTGTGACTC

TCATAACTGCCGTCTGTTCGTTCTCTGCCCATCTCTTGCCCGTGGAGCTCTGTCGAGGCCAACCCAGCCAAGGGGCCGGGTTACCCACT

CATCTGCTCCAACTGCCTTCCCAGATCTCCCTTCTCCCTCCTTCTATGGAGTGAGGGTCCAGGGGCCTGCCCAGCACCAGCCTGTGCTC

CTGCGAATTTCTGCTCTCTGAGGTCTCCTCGCTCCTTTTCCCATCTGGGTGTTG|AGATCGGAAGAGCGTCGTGTAGGGAAAGAGTGTT

AAGATTAGTGTAGATCTCGGT
```
